# Parp1 Localizes within the *Dnmt1* Promoter and Protects Its Unmethylated State by Its Enzymatic Activity

**DOI:** 10.1371/journal.pone.0004717

**Published:** 2009-03-05

**Authors:** Michele Zampieri, Claudio Passananti, Roberta Calabrese, Mariagrazia Perilli, Nicoletta Corbi, Fabiana De Cave, Tiziana Guastafierro, Maria Giulia Bacalini, Anna Reale, Gianfranco Amicosante, Lilia Calabrese, Jordanka Zlatanova, Paola Caiafa

**Affiliations:** 1 Department of Cellular Biotechnologies and Hematology, Second Faculty of Medicine, University “La Sapienza”, Rome, Italy; 2 Department of Biochemical Sciences, University “La Sapienza”, Rome, Italy; 3 Pasteur Institute-Fondazione Cenci Bolognetti, Rome, Italy; 4 Institute of Molecular Biology and Pathology CNR, Rome, Italy; 5 Department of Biomedical Sciences and Technologies, University of L'Aquila, L'Aquila, Italy; 6 Department of Molecular Biology, University of Wyoming, Laramie, Wyoming, United States of America; University of Arkansas for Medical Sciences, United States of America

## Abstract

**Background:**

Aberrant hypermethylation of CpG islands in housekeeping gene promoters and widespread genome hypomethylation are typical events occurring in cancer cells. The molecular mechanisms behind these cancer-related changes in DNA methylation patterns are not well understood. Two questions are particularly important: (i) how are CpG islands protected from methylation in normal cells, and how is this protection compromised in cancer cells, and (ii) how does the genome-wide demethylation in cancer cells occur. The latter question is especially intriguing since so far no DNA demethylase enzyme has been found.

**Methodology/Principal Findings:**

Our data show that the absence of ADP-ribose polymers (PARs), caused by ectopic over-expression of poly(ADP-ribose) glycohydrolase (PARG) in L929 mouse fibroblast cells leads to aberrant methylation of the CpG island in the promoter of the *Dnmt1* gene, which in turn shuts down its transcription. The transcriptional silencing of *Dnmt1* may be responsible for the widespread passive hypomethylation of genomic DNA which we detect on the example of pericentromeric repeat sequences. Chromatin immunoprecipitation results show that in normal cells the *Dnmt1* promoter is occupied by poly(ADP-ribosyl)ated Parp1, suggesting that PARylated Parp1 plays a role in protecting the promoter from methylation.

**Conclusions/Significance:**

In conclusion, the genome methylation pattern following PARG over-expression mirrors the pattern characteristic of cancer cells, supporting our idea that the right balance between Parp/Parg activities maintains the DNA methylation patterns in normal cells. The finding that in normal cells Parp1 and ADP-ribose polymers localize on the *Dnmt1* promoter raises the possibility that PARylated Parp1 marks those sequences in the genome that must remain unmethylated and protects them from methylation, thus playing a role in the epigenetic regulation of gene expression.

## Introduction

5-methylcytosine is considered to be the fifth base of DNA as – through its non-random distribution along the genome – it constitutes part of the epigenetic chromatin modifications that control gene expression patterns. The genome methylation pattern is bimodal: the methylated cytosines are scattered throughout the genome, whereas the unmethylated residues are mainly located within particular regions termed CpG islands (CGIs) [1]–[3]. The 37,000 CGIs in the mouse genome represent 1–2% of the DNA and are generally located in the 5′ promoter regions of the housekeeping genes, sometimes overlapping the coding region to variable extents. Although their sequence is enriched in CpG dinucleotides, the best substrates for DNA methyltransferase activity, the CGIs are mainly unmethylated and the associated genes are actively transcribed; transcription is inhibited when these regions undergo methylation [4]–[7].

In cancer cells, there are drastic changes in the DNA methylation patterns: the housekeeping gene promoters become hypermethylated, whereas the genome as a whole undergoes significant hypomethylation events. The mechanisms by which CGIs are protected from methylation in both replicating and non-replicating chromatin in *normal* cells, and the mechanism(s) whereby these DNA regions become susceptible to methylation in *tumor* cells are still unknown [2], [8], [9]. The inversion of DNA methylation patterns observed on inactive X *vs* active X chromosomes is also far from understood [2].

A significant amount of research has been carried out over the years to see if the levels of Dnmt1 control the aberrant methylation pattern in tumor cells and in cells where Dnmt1 was stably overexpressed [10], [11]. Indeed, *Dnmt1* silencing allows demethylation and re-expression of some germ-line specific genes whose repression is methylation-dependent in somatic cells [12], [13]. The promoters of these genes become demethylated also in many tumor cells, opening up the possibility that passive demethylation, due to silencing of *Dnmt1*, is involved in determining the diffuse genome-wide hypomethylation which has been associated with chromatin decondensation [8], genomic instability [14], apoptosis [15], cancer [4], [6], [7], [16], disruption of nucleolar architecture [17], aberrant telomere elongation [18], loss of imprinting during preimplantation development [19], [20], and even mitotic catastrophe [21].

Over the past decade our laboratory has accumulated evidence that links poly(ADP-ribosyl)ation with DNA methylation, suggesting that poly(ADP-ribosyl)ation is involved in maintaining DNA methylation patterns. A series of different experimental strategies suggests that blockage of poly(ADP-ribosyl)ation, due to competitive inhibition of poly(ADP-ribose) polymerases (PARPs), induces *in vivo* DNA hypermethylation, both on genomic DNA [22]–[24] and on particular CGI regions [25]. On the other hand, cells with hyperactive Parp1 are characterized by a widespread DNA hypomethylation [26]. We have suggested a mechanism in which Parp1 in its automodified (PARylated) form or PARs themselves make Dnmt1 catalytically inactive and, thus, inefficient in DNA methylation [27]. In this model, modified Parp1, through the high negative charge of bound PARs attracts and hosts Dnmt1, thus preventing its catalytic activity. In fact, we found that Dnmt1 possesses two presumptive PAR-binding domains and shows higher affinity for free polymers than for DNA. Co-immunoprecipitation data indicated that Dnmt1 and Parp1 associate *in vivo* and that the Parp1 present in the complex is in its PARylated form [27].

We hypothesize that the right nuclear balance between unmodified and PARylated forms of Parp1 – which depends on the correct dynamics of Parp/Parg activities – determines the maintenance of DNA methylation patterns [28]. According to our data, decreased or increased levels of PARylated Parp1 are responsible for diffuse hypermethylation or hypomethylation of DNA, respectively. In the absence of PARylated Parp1, Dnmt1 is free to methylate DNA; conversely, under conditions of persistently high levels of PARylated Parp1, the stable inhibition of Dnmt1 would prevent its methylation-maintenance activity at replicative forks, thus leading to passive DNA hypomethylation of the genome.

These findings underscore the importance of a rapid reversal of Parp1 automodification since it affects the epigenetic information. They also suggest that the introduction of new methyl groups onto CGIs of housekeeping genes and/or the diffuse genome hypomethylation in cancer cells could also occur through deregulation of Parp or Parg activities.

In this work, the non-specific effects of inhibitors of Parp activity were excluded by using ectopic over-expression of PARG to deplete cells of PARs. We show that following over-expression of PARG: i) *Dnmt1* expression is down-regulated; ii) the CGI in the promoter of *Dnmt1* loses its protection against methylation and becomes methylated; iii) in normal cells, Parp1 and PARs locate on the *Dnmt1* minimal promoter; iv) the silencing of the *Dnmt1* gene is accompanied by diffuse demethylation of the genome, including the pericentromeric repeat sequences which are methylated in normal cells. These findings suggest that Parp1 occupies the *Dnmt1* promoter and protects its unmethylated state through its automodification activity, i.e. its ability to build poly(ADP-ribose) chains onto itself.

## Results

### Myc-PARG localizes in the nucleus of transfected L929 mouse fibroblasts and degrades endogenous PARs

The complete coding region for human PARG was cloned into the Myc-tag expression vector pCS2-MT and the expression of the Myc-PARG protein was evaluated in transfection assays in the mouse fibroblast cell line L929.

**Figure 1:**
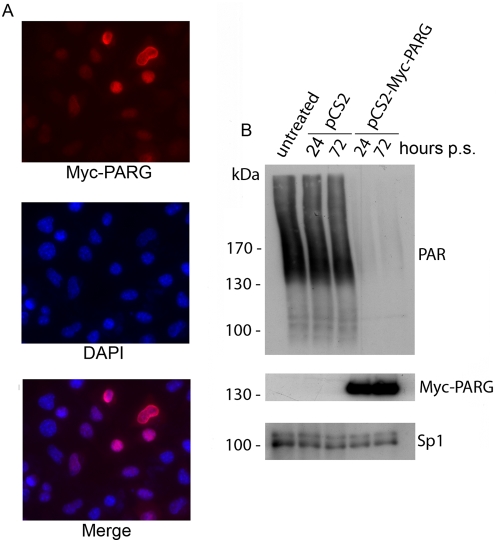
Ectopic over-expression of PARG. A, Immunofluorescence images showing the nuclear localization of ectopically over-expressed PARG. B, Western blot of proteins from cell cultures at 24 and 72 hours of puromycin selection (p.s.) transfected with pCS2-Myc-PARG vector 24 hours before selection *vs* control cells. Analyses were performed using anti-PAR antibodies, anti-Myc epitope antibody for Myc-PARG; anti-Sp1 antibody was used as endogenous control. pCS2: empty vector; pCS2-Myc-PARG: vector containing full length cDNA for human PARG.

Figure 1A shows a predominant nuclear localization of Myc-PARG at 48 hours of transient transfection, as evaluated by immunofluorescence analysis. Western blot experiments performed on nuclear lysates of over-expressing cells show that the level of Myc-PARG, (which is stable up to 72 hours of puromycin selection, Figure 1 B, middle panel), introduces a sharp decrease in PARs, when compared to PARs level either in non-transfected cells or in cells transfected with empty vector (Figure 1B, upper panel). These results clearly demonstrate the capability of Myc-PARG to act on endogenous substrates, causing an almost complete disappearance of PARs.

With the aim of evaluating the effect of Myc-PARG over-expression on cell viability, we measured the percentage of live cells by the trypan blue-exclusion assay at 24 and 72 hours of puromycin selection. Although we observed a significant reduction in the number of live cells within each sample between 24 and 72 hours of selection, the survival level in the Myc-PARG over-expressing cultures was not affected (if anything, it slightly increased in comparison with the control cultures) (Figure S1 A). The evaluation of lactate dehydrogenase (LDH) activity in the culture medium of transiently transfected cultures revealed that the cytotoxicity depended on the transfection procedure, and not on the Myc-PARG over-expression (Figure S1 B). Methods used for determination of cell viability, cell-cycle progression and cytotoxicity are available as supporting information (Materials and Methods S1).

### *Dnmt1* expression is down-regulated in Myc-PARG over-expressing L929 cells

**Figure 2:**
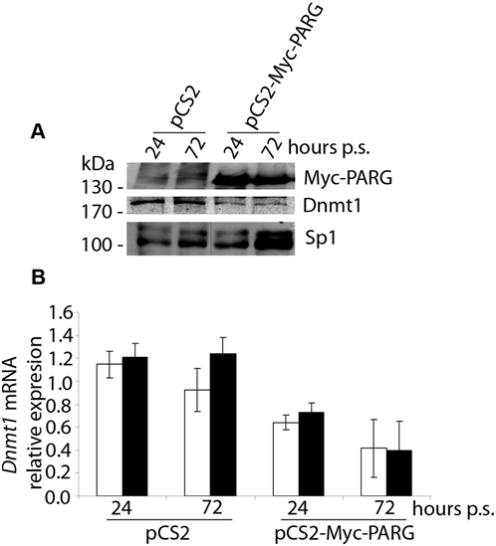
Ectopic over-expression of PARG leads to down-regulation of Dnmt1 expression. A, Nuclear lysates from cultures transfected with pCS2 and pCS2-Myc-PARG vectors at 24 and 72 hours of puromycin selection were analyzed by SDS-PAGE and Western blot using anti-Myc epitope and anti-Dnmt1 antibodies; anti-Sp1 antibodies served as endogenous control. B, Real time RT-PCR for *Dnmt1* performed on RNA samples from cell cultures transfected with pCS2 and pCS2-Myc-PARG vectors at 24 and 72 hours of puromycin selection. *Gapdh* (white bar) and *Hprt1* (black bar) mRNAs were used as endogenous controls. Data are reported as mean±S.E. of the ratio of *Dnmt1* mRNA level to *Gapdh* or *Hprt1* mRNA level within each sample, calculated from a minimum of three experiments performed in duplicate.

Western blot experiments (Figure 2A) carried out on nuclear lysates show that the Dnmt1 protein level decreases after Myc-PARG over-expression. Real-time RT-PCR experiments show that this reduction depends on down-regulation of *Dnmt*1 mRNA level (Figure 2B), suggesting a regulatory role of PAR in *Dnmt*1 gene transcription.

As the expression level of *Dnmt1* is cell-cycle dependent, we checked the cell cycle progression by FACS analysis. We excluded any cytostatic effect of Myc-PARG over-expression under the adopted experimental conditions, as we did not detect significant differences between samples except for the sub-G1 fraction, which could be due to puromycin selection (Figure S2).

### Myc-PARG-mediated PAR depletion disrupts the methylation pattern of the CGI located in 5′ regulatory region of *Dnmt*1

**Figure 3:**
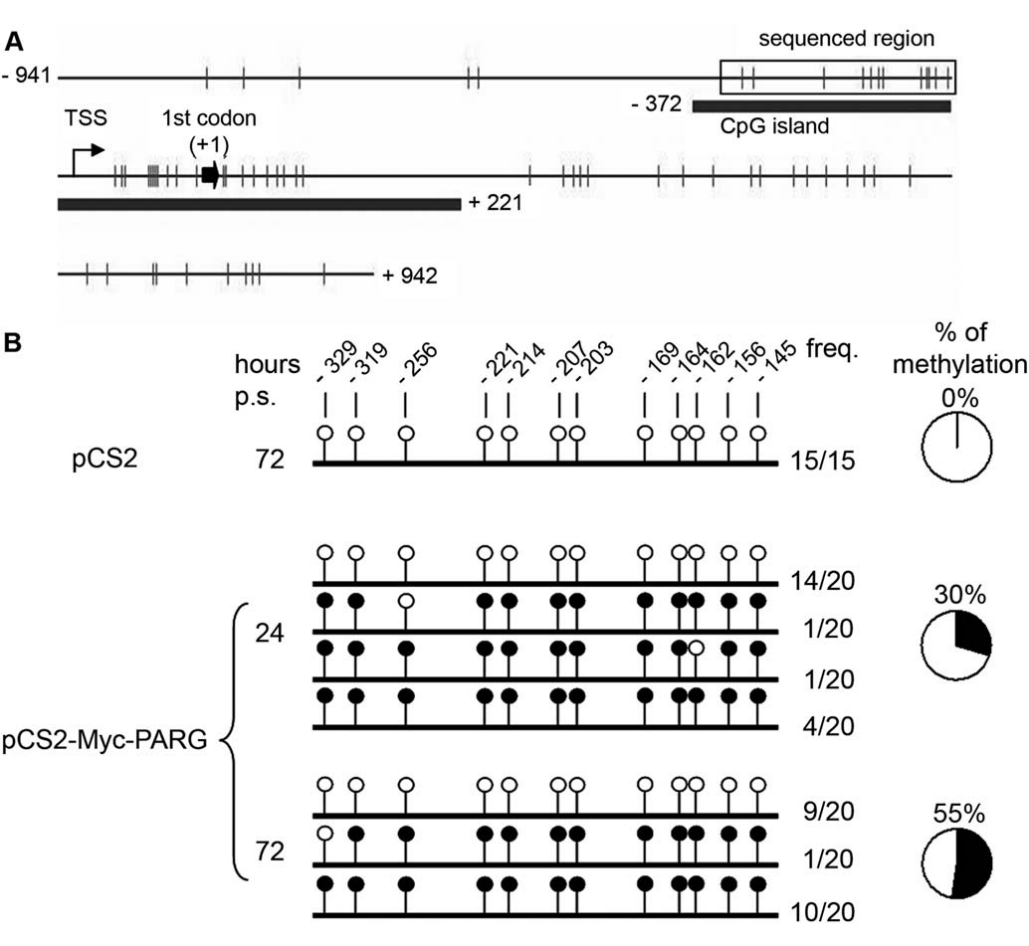
Ectopic over-expression of PARG changes the methylation pattern of the CGI located in 5′ regulatory region of the *Dnmt1* gene. A, Graphical representation of the CGI region (gray line) in the promoter region of *Dnmt1*. Numbers indicate the distance in base pairs from the first codon (black arrow); TSS: transcription start site; dashes: GpG dinucleotides. B, The methylation state of *Dnmt1* promoter region spanning base pairs −364 to −103 was evaluated by bisulphite sequencing following transfection with either pCS2 or pCS2-Myc-PARG vectors at 24 and 72 hours of puromycin selection. Up to twenty independent clones for each sample were analysed by the sequencing procedure. Each row of circles represents the sequence of an individual clone. Open circle, unmethylated CpG site; filled circle, methylated site. Frequency: number of clones with depicted methylation pattern of all clones tested under the specified conditions.

Based on our previous data demonstrating the introduction of anomalous methyl groups onto some CGIs upon competitive inhibition of Parp activity [25], we hypothesized that the down-regulation of *Dnmt*1 gene expression observed in PARG over-expressing cells is associated with changes in the methylation pattern of its promoter. By bisulphite sequencing we determined the methylation pattern of a portion of the CpG island in the *Dnmt*1 promoter region in close proximity to the transcription start site (Figure 3A). Methylation pattern analysis was performed on 15–20 clones for each sample. The twelve CpG dinucleotides, present in the sequence under examination, were found methylated in ∼30% of the clones at 24 hours of puromycin selection (48 hours from transfection), with ∼55% of the clones methylated at 72 hours of puromycin selection (96 hours from transfection). These 12 CpG dinucleotides are unmethylated in the control sample (Figure 3B). The increased percentage of methylated clones may reflect the increased clearing of untransfected cells by puromycin at the longest selection time (Figure S1A).

### PARs and Parp1 localize at the *Dnmt1* minimal promoter

**Figure 4:**
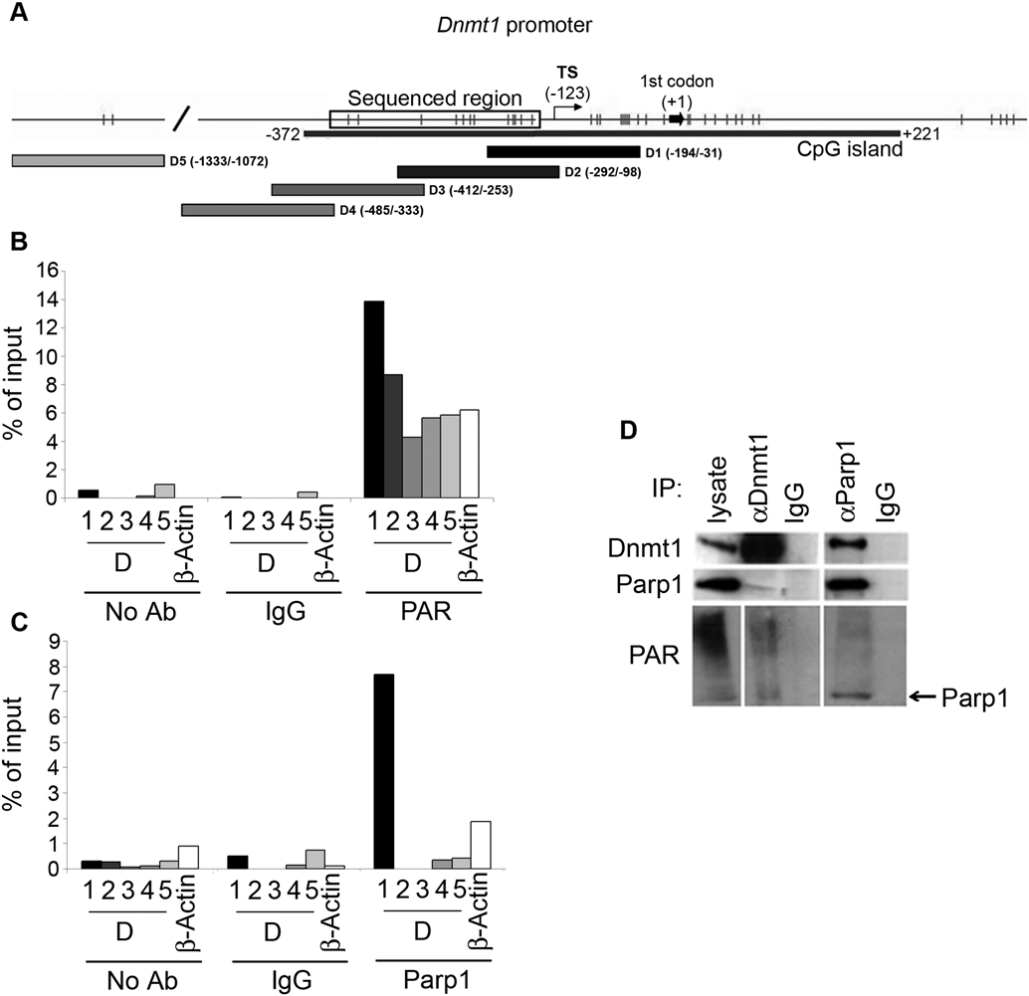
ChIP analysis of *Dnmt1* promoter occupancy by PARs and Parp1. A, Schematic representation of the *Dnmt1* promoter region with approximate locations of the amplicons used to detect the presence of *Dnmt1* sequences in ChIP complexes. ChIPs were carried out with anti-PAR (B) and anti-Parp1 (C) antibodies. Controls were non-specific normal rabbit IgGs (IgG) or no antibody (No Ab). DNA was amplified by real-time PCR with primer sets for the amplicons indicated in A; a primer set for the *β-actin* promoter was used as control. Numbers refer to distance in base pairs from the first codon. Data are expressed as percentage of the signal detected for the non-immunoprecipitated input (4% of the chromatin subjected to immunoprecipitation) taken as 100%. D, Western Blot analysis of samples immunoprecipitated with either anti-Parp1 or anti-Dnmt1 antibodies; anti-PAR antibody was used to detect polymers in the immunoprecipitated complexes.

Chromatin immunoprecipitation (ChIP) experiments were performed to verify if PARs and Parp1 colocalize on the *Dnmt1* promoter to prevent its methylation in normal cells. Cross-linked chromatin was immunoprecipitated with antibodies anti-PARs and anti-Parp1. The region of DNA of about 1000 bp – covering the proximal promoter region (amplicons D1–D4) or the distal region (D5) – was probed and the presence of each fragment was evaluated by quantitative PCR (Figure 4A). Primers specific for the *β-actin* promoter were used as control. ChIP analysis, carried out with anti-PAR antibodies, showed that PARs are particularly enriched within the region spanning from −31 to −292 bp (primers D1 and D2), overlapping the *Dnmt1* minimal promoter located within 300 bp from the first codon [29]. Conversely, the more distal regions did not show any enrichment in PARs when compared with the control *β-actin* promoter (Figure 4B). As it is well known that Parp1 is responsible for more than 90% of PAR synthesis in cells, we performed ChIP analysis with antibodies against Parp1 to assess whether Parp1 could harbour PARs at the *Dnmt1* promoter. Figure 4C shows that Parp1 specifically localizes within the region amplified by D1 primers, which was found to be also highly enriched in PARs. Significant positive signals were not detected for the other *Dnmt1* promoter regions under consideration or for the *β-actin* promoter control. Immunoprecipitation experiments, carried out with both anti-Parp1 and anti-Dnmt1 antibodies, confirmed earlier results [26], [27] that the two proteins interact *in vivo* and that Parp1 is PARylated in the complex (Figure 4D).

Our attention was then focused on the highly conserved and ubiquitously expressed nuclear factor Ctcf [30], as this protein, which is one of the major players in imprinting and insulator processes [31], [32], puts together the two epigenetic events we are interested in: poly(ADP-ribosyl)ation [33], [34] and DNA methylation [35]–[37]. Thousands of Ctcf binding sites have been identified in recent genome-wide localization studies, and their distribution along the genome further supports a crucial role of Ctcf as a chromatin organizer [38]–[41].

**Figure 5:**
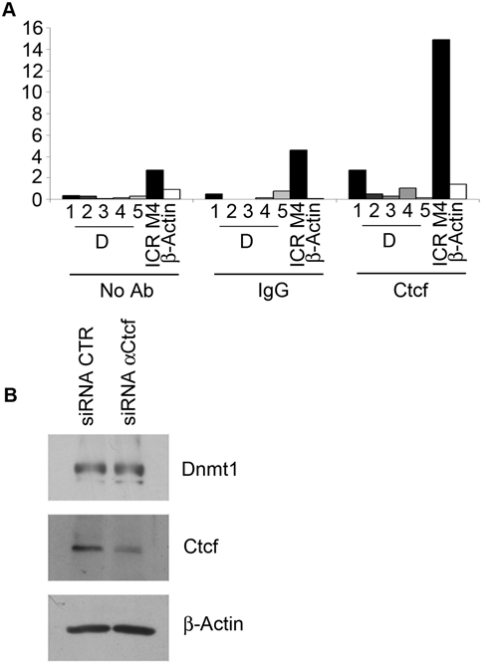
ChIP analysis of *Dnmt1* promoter occupancy by Ctcf. A, ChIP was carried out with anti-Ctcf antibodies. The imprinting control region ICR M4 (*Igf2/H19* locus) was used as a positive control for Ctcf binding (For further specifications about control ChIPs and the set of primers used see legend to figure 4). B, Western Blot analysis of total cell lysates from L929 cells transfected with anti-Ctcf siRNA and detected with anti-Ctcf and anti-Dnmt1 antibodies. β-Actin served as endogenous control.

Our previous data showed that Parp1 becomes PARylated when it interacts with Ctcf, i.e. Ctcf activates Parp1 [26]. Therefore, Ctcf is an important player in the Parp1/Dnmt1 interplay since PARylated Parp1 plays an inhibitory role on Dnmt1 activity [26]. ChIP assays with antibodies against Ctcf were performed to verify if Ctcf interacting with Parp1 is responsible for the presence of PARylated Parp1 at the *Dnmt1* promoter. The data presented in Figure 5A indicate that Ctcf was not present on the *Dnmt1* promoter. The involvement of Ctcf in the maintaining of the unmethylated state of *Dnmt1* promoter was further disproved by Ctcf siRNA-silencing assays where *Dnmt1* expression was not affected by a decreased level of Ctcf (Figure 5B).

### Myc-PARG over-expression affects the DNA methylation machinery

**Figure 6:**
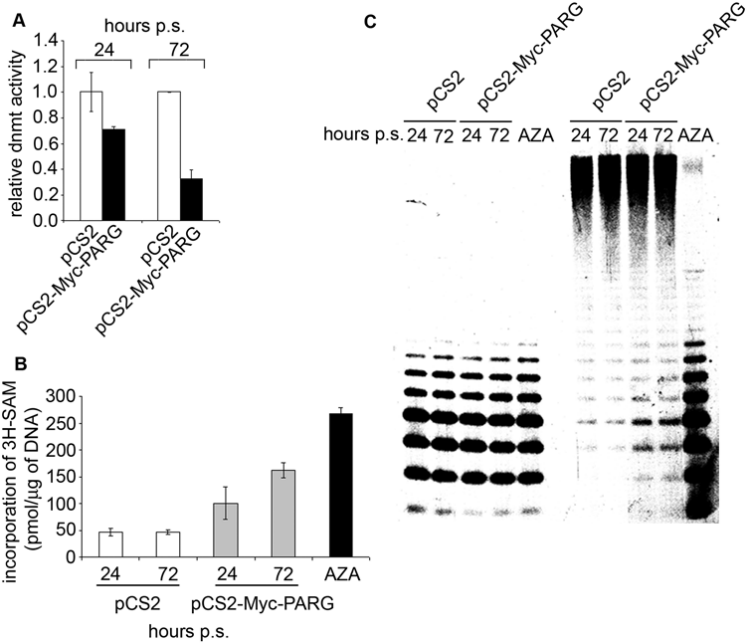
Dnmt1 down-regulation dependent on PARG over-expression leads to a widespread genome hypomethylation. A, Endogenous DNA methyltransferase activity (dnmt) of nuclear extract from cultures at 24 and 72 hours of puromycin selection transfected with either pCS2 (white bar) or pCS2-Myc-PARG (black bar) vectors. The DNA methyltransferase activity of pCS2 samples was considered as 1.0. B, Methyl-accepting ability assay was carried out on genomic DNA purified from cells transfected with either pCS2 (white bars) or pCS2-Myc-PARG (black bars) vectors at 24 and 72 hours of puromycin selection. Results are displayed as number of picomoles of labelled S-Adenosyl methionine incorporated per microgram of DNA. DNA obtained from cells treated with 5-AZA was used as positive control for genome hypomethylation (black bar). Data reported in A and B are mean±S.E. of three experiments, each performed in triplicate. C, Analysis of Southern blot against minor satellite DNA repeats performed on genomic DNA purified from cells transfected with either pCS2 or pCS2-Myc-PARG vectors at 24 and 72 hours of puromycin selection and digested with HpaII or MspI restriction enzymes. DNA obtained from cells treated with 5-AZA was used as positive control for genome hypomethylation.

The down-regulation of *Dnmt1* expression occurring as a result of Myc-PARG mediated PAR degradation prompted us to look for effects on the DNA methylation machinery and the genome-wide methylation patterns. The ability of nuclear lysates to methylate exogenous DNA is gradually compromised after transfection of Myc-PARG (Figure 6A). These data correlate with results of DNA methyl-accepting ability assays showing a widespread hypomethylation of DNA extracted from cells over-expressing PARG (Figures 6B). The enhanced incorporation of exogenous labelled methyl groups on this DNA *vs* the respective controls reveals that the genome underwent demethylation (the demethylated DNA has higher DNA methyl-accepting ability). Analyses of the methylation state of methyl-CpG rich centromeric minor satellite DNA repeats, assayed by the methyl-sensitive restriction enzyme HpaII, show that new unmethylated cutting-prone sites are formed in these sequences when Myc-PARG is overexpressed, resulting in enhanced fragmentation (Figure 6C).

## Discussion

The data reported here indicate that, following ectopic over-expression of PARG, the promoter of *Dnmt1* is no longer protected from anomalous methylation; moreover, the insertion of new methyl groups onto the CpG island in the promoter region leads to transcriptional down-regulation of the gene. We have earlier shown that PARs - either protein free or bound to PARylated Parp1 - compete with DNA for binding to Dnmt1; when the enzyme is hosted on the polymers, it can no longer perform its catalytic function on DNA [27]. Here, we suggest that the CGI in the promoter of *Dnmt1* is protected from methylation by PARylated Parp1 or a PARylated transcriptional factor which attracts Dnmt1 and inhibits its activity. By chromatin immunoprecipitation experiments using anti-PAR and anti-Parp1 antibodies, we showed that the *Dnmt1* promoter region that was methylated following PARG over-expression, contained both Parp1 and PARs in control (non-over-expressing) cells. We can conclude that in normal cells PARylated Parp1 (or a PARylated transcription factor) occupies the *Dnmt1* promoter, maintaining this region unmethylated.

To establish whether PARylated Parp1 by itself can regulate the promoter region of *Dnmt1* gene, we focused our attention on the transcriptional factor Ctcf. We excluded the involvement of Ctcf in this mechanism as: (i) the *Dnmt1* promoter could not be chromatin immunoprecipitated with anti-Ctcf antibody, and (ii) decreased levels of Ctcf did not affect the level of Dnmt1. We cannot exclude that Parp1 is recruited to the *Dnmt1* promoter by other transcription factors. The predictive Transfac search for transcription factor binding sites within the promoter region amplified by D1 and D2 primers revealed multiple putative binding sites for transcription factors, some of which are known to interact with Parp1. In particular, the promoter region from −31 bp to −64 bp from the first codon, which is specifically amplified by D1 primers, contains predicted binding sites for p300, SRY, and E2F transcription factors, recognized Parp1 partners [42]–[44].

In the absence of PARs, the molecular mechanism that protects the unmethylated state of the *Dnmt1* promoter is lost, with consequent introduction of new methyl groups onto the promoter CpGs, and silencing of the gene. As Dnmt1 is one of the most important players in maintaining the genome methylation pattern during DNA replication, the silencing of the gene in turn leads to widespread passive hypomethylation.

In conclusion, the deregulation of PAR levels, dependent on the balance between PARP and PARG activities, may lead to reversal of the normal methylation pattern by (i) introducing aberrant methylation of some normally unmethylated CGIs (e.g. *Dnmt1* CGI) and (ii) causing genome-wide hypomethylation. The changes in the DNA methylation patterns caused by over-expressing PARG, i.e. by persistently low levels of polymers, mimic the epigenetic changes that occur in cancer, where there is hypermethylation of the CGI promoter regions of tumor suppressor genes and widespread genomic DNA hypomethylation [45].

Experiments with PARG over-expression reveal an additional mechanism responsible for the widespread genomic hypomethylation in cancer cells. In the previously established mechanism *high levels* of PARylated PARP-1 inhibit Dnmt1 activity by hosting the enzyme on the poly(ADP-ribose) polymers [26]. The new mechanism, on the other hand, functions under conditions of polymer levels *too low* to inhibit Dnmt1 activity; in these conditions, new methyl groups are inserted onto the *Dnmt1* promoter, which leads to down-regulation of the gene. Thus, poly(ADP) ribose levels that are either too high or too low lead to the same outcome in terms of the global methylation status of the genome: they both cause widespread genome hypomethylation. These results underscore once again the critical importance of keeping balanced polymer levels, as we have recently argued [28].

Recently, Parp has been implicated in multiple pathways that regulate gene expression, including effects on chromatin structure [46], [47] and transcriptional activator and coactivator functions [48]. Our findings add another dimension to the regulatory functions of Parp, through affecting DNA methylation patterns. A new question now emerges: does PARylated Parp1 introduce an epigenetic mark on chromatin? PARylated Parp1 could mark those DNA sequences that must be maintained in a non-methylated state in normal cells and directly prevent Dnmt1 access to these sequences.

## Materials and Methods

### Cell culture and drug treatment of cells

L929 mouse fibroblasts were maintained as sub-confluent culture in high glucose (4.5 g/litre) Dulbecco's modified Eagle's medium, supplemented with 10% fetal calf serum, 2 mM L-glutamine, 50 units/ml Penicillin and 50 µg/ml Streptomycin. All culture solutions were from International PBI.

To obtain hypomethylated DNA, cells were cultivated for 72 hours in standard medium containing 5 µM 5-azacytidine (5-AZA) (Sigma).

### Plasmids construction and transfection of cells

Human PARG cDNA, containing the complete coding region, was isolated by PCR amplification using as template cDNA prepared from poly A+ selected RNA from human adult skeletal muscle. The following oligonucleotides were used in the PCR reaction: 5′-CCGGAATTCAATGAATGCGGGCCCCGGCTGTGAACCC-3′ sense, -5′-GCCGCTC GAGTCAGGTCCCTGTCCTTTGCCCTGAATG-3′ antisense. The amplified DNA fragment was cloned in the Myc-tag expression vector pCS2-MT and sequenced by GeneLab Service (Enea-Casaccia).

In transfection experiments 0.5×10^6^ cells were seeded in 60×15 mm culture dishes (Greiner bio-one) and transfected with Lipofectamine Plus reagent (Invitrogen) adopting the manufacturer's protocol. Assays were performed with 4 µg/dish of purified plasmid DNA of either empty myc-vector (pCS2) as control or Myc–PARG construct (pCS2-Myc-PARG) together with 0.4 µg/dish of pBabe-puro (Addgene) vector for puromycin selection of transfected cells. After 24 hours cells were incubated for further 24 or 72 hours in culture medium supplemented with puromycin (2 µg/ml, Calbiochem). Apart from seeding 0.25×10^6^ cells/dish and omitting pBabe-puro and puromycin selection, the same procedure was employed in transient transfection assays.

### Western blot analysis

Nuclei were collected from trypsinized and phosphate-buffered saline (PBS)-washed cells by centrifugation following incubation (30 minutes) in isolation buffer containing 10 mM Tris-HCl pH 7.9, 4 mM MgCl_2_, 1 mM EDTA, 0.5 mM dithiothreitol, 0.25 mM sucrose, 1% Triton X-100. Nuclear fraction was lysed in RIPA buffer (50 mM Tris-HCl pH 8, 150 mM NaCl, 0.5% sodium deoxycholate, 0.1% SDS, 1% Nonidet P-40, 1 mM EDTA). Both buffers were supplemented with protease inhibitors (complete EDTA-free, Roche Applied Science). Protein concentration was determined using the Bradford protein assay reagent (Bio-Rad) with bovine serum albumin (Promega) as standard. Equal protein amounts were subjected to 8% SDS-PAGE and blotted onto Hybond-ECL nitrocellulose membranes (Amersham Biosciences). The antibodies employed were as follows: mouse monoclonal Ab anti-PAR (10 HA, Trevigen), mouse monoclonal Ab anti-Myc (9E10 clone, hybridoma-conditioned medium), mouse monoclonal Ab anti-Dnmt1 (Imgenex), mouse monoclonal Ab anti-β-Actin (Sigma), mouse monoclonal Ab anti-Parp1 (C2-10, Alexis), rabbit polyclonal Ab anti-Ctcf (Upstate), rabbit polyclonal Ab anti-Sp1 (H-225, Santa Cruz Biotechnology), and goat antimouse and anti-rabbit horseradish peroxidase-conjugated antibodies (Santa Cruz Biotechnology).

### Immunofluorescence

Immunofluorescence was performed to detect Myc-PARG. Transiently transfected L929 cells (at 48 hours following transfection) were fixed and permeabilized in methanol/acetone mixture 3∶7, for 20 min at RT and then incubated for 1 hour with anti-myc hybridoma conditioned medium (9E10). Bound antibody was visualized using Alexa Fluor-rhodamine-conjugated anti-mouse Ig secondary antibody (Molecular Probes). Nuclei were visualized by staining with Hoechst (1 mg ml^−1^) (Sigma). Stained samples were examined by conventional epifluorescence microscopy (Olympus BX51; Tokio, Japan).

### Extraction of nucleic acids

Plasmid DNA and genomic DNA were prepared by Plasmid Maxi Kit and DNeasy tissue kit respectively (Qiagen). Total RNA was purified by RNeasy mini kit (Qiagen). Concentration, purity and integrity of preparations were evaluated spectrophotometrically, followed by agarose gel-ethidium bromide electrophoresis.

### qRT-PCR

Total RNA (1 µg) was subjected to retrotrascription using Superscript First-Strand Synthesis system (Invitrogen). Expression of mRNA for *Dnmt1* was measured by real time PCR using Taq-Man gene expression assays (Applied Biosystems) following the manufacturer's protocol for the absolute standard curve method on iCycler IQ detection system (Bio-Rad). The standard curve was generated using 1∶1 serial dilutions (from 100 to 12.5 ng) of cDNA obtained from control cells at 24 hours as reference. PCR efficiency was 90–100% for each set of primers and probe in any experiment. The amplification reaction was performed in duplicate for each sample in 96-well plates. The amount of *Dnmt1* mRNA was calculated adopting the standard curve method, and normalization was carried out using hypoxanthine-guanine phosphoribosyltransferase (*Hprt1*) and glyceraldehyde-3-phosphate dehydrogenase (*Gapdh*) as internal control genes. TaqMan gene expression assay IDs for each set of primers and probe were as follows: Mm00599763m1 (*Dnmt1*); Mm00446968m1 (*Hprt1*) and Mm99999915g1 (*Gapdh*).

### Chromatin immunoprecipitation (ChIP)

Subconfluent L929 cell cultures (about 1×10^5^ cells/cm^2^ in standard culture dishes) were crosslinked at room temperature for 15 min by 1% formaldehyde (Fluka) in normal medium. Reaction was stopped by 5 min incubation in 0.125 M Glycine (Sigma) in PBS. Cell monolayer was harvested by scraping in ice-cold PBS containing protease inhibitors. Cell lysis was performed in 1% SDS, 10 mM EDTA, 50 mM Tris-HCl pH 8.0, protease inhibitors. Soluble fraction from 10^6^ cells was isolated by centrifugation at 13.000×g for 15 min at 4°C. Lysate – 100 µl per assay - was pre-cleared by addition of 0.9 ml of dilution buffer (0.01% SDS, 1.1% Triton X-100, 1.2 mM EDTA, 16.7 mM Tris-HCl pH 8.0, 167 mM NaCl, protease inhibitors) and 45 µl of protein A–Sepharose pre-blocked with salmon sperm DNA (Upstate). A 40 µl aliquot corresponding to 4% of precleared lysate was taken as input control. Pre-cleared lysates were then incubated with specific antibodies (2.5 µg) overnight at 4°C. Control immunoprecipitations without antibody (No Ab) and with purified normal rabbit total IgGs (Santa Cruz) were also performed. Immunocomplexes were recovered from lysates by incubation at 4°C for 2 hours with 45 µl of protein A–Sepharose pre-blocked with salmon sperm DNA (Upstate). Precipitates were successively washed (10 min each wash) with 1.0 ml of the following buffers: low salt (0.1% SDS, 1% Triton X-100, 2 mM EDTA, 20 mM Tris-HCl pH 8.0 , 150 mM NaCl), high salt (0.1% SDS, 1% Triton X-100, 2 mM EDTA, 20 mM Tris-HCl pH 8.0, 500 mM NaCl), LiCl (250 mM LiCl, 1% Nonidet P-40, 1% Na-deoxycholate, 1 mM EDTA, 10 mM Tris-HCl pH 8.0). All wash buffers had protease inhibitors added. Following two final washes in TE (10 mM Tris-HCl pH 8.0, 1 mM EDTA), the immunocomplexes were finally eluted in 150 µl of TE/1% SDS buffer (10 mM Tris-HCl pH 8.0, 5 mM EDTA, 1% SDS) by incubation at 65°C for 15 min. The formaldehyde cross-link was reversed by incubating the sample at 65°C overnight; the input sample was processed in parallel with ChIP samples from this point on. DNA fraction was recovered by proteinase K (Roche) digestion followed by phenol-chloroform extraction and ethanol precipitation in the presence of glycogen. DNA pellets were resuspended in 200 µl of water. Real Time PCR reactions were carried out in a final volume of 25 µl of iQ SYBR Green Supermix (Bio-Rad), with 150 nM specific primers and 5 µl of DNA added. PCR amplification was performed on a iCyclerIQ (Bio-Rad) thermal cycler adopting the following conditions: the initial denaturation at 95°C for 10 min; 45 cycles of: 95°C for 30 sec, 60°C for 30 sec, and 72°C for 30 sec. The threshold cycle (CT) values of ChIP signals detected by real-time PCR were converted to the percentage of each ChIP signal for input DNA, which were calculated by the delta–delta method according to the following equation: sample signal = 100×[2^(CT IP sample–CT Input)^].

Antibodies used for immunoprecipitations were the following: mouse monoclonal anti-poly(ADP-ribose) polymer (10 HA, Trevigen); rabbit polyclonal anti-poly(ADP-ribose) polymerase 1 (Alexis); rabbit polyclonal anti-Ctcf (Upstate).

Primers used in real-time PCR assays were the following:

D1 sense 5′-TATAGCCAGGAGGTGTGGGTG-3′;

 antisense 5′AACGAGACCCCGGCTTTTT-3′;

D2 sense 5′-TCCTCTGCAAGAGCAGCACTA-3′;

 antisense 5′- ATGTACCACACAGGGCAAGA-3′;

D3 sense 5′-TGTTTGTGCATGTGAGTGCA-3′;

 antisense 5′-TCGGCACTTGAGAGCAGGTA-3′;

D4 sense 5′-TGAGTGCTGGAATCAAATGC-3′;

 antisense 5′-AAGCCCCTGTAATTCCACTT-3′;

D5 sense 5′-AGAAGTGGTTCCTGGCCTTA-3′;

 antisense 5′-TAACTCTATCCCCCTCCCCTT-3′;

β-Actin sense 5′-TTGGCTCCGCGTCGCTCACTCAC-3′;

 antisense 5′-CCCCAGAATGCAGGCCTAGTAACCGAGAC-3′;

ICR M4 sense 5′-CAATGATTCATAAGGGTCAT-3′;

 antisense 5′-CGTAAGTGCACAAATGCC-3′.

### DNA methyltransferase activity

Equal amounts of nuclear lysates were analyzed for DNA methyltransferase activity by the EpiQuikTM DNA methyltransferase assay kit (Epigentek) following the manufacturer's conditions.

### Methyl-accepting ability assay

Methyl-accepting ability assay was carried out in a final volume of 50 µl of 10 mM Tris-HCl pH 7.9, 10 mM MgCl2, 50 mM NaCl, 1 mM dithiothreitol in the presence of 1 µg of purified DNA and 1 unit of bacterial SssI methylase (New England Biolabs), using as methyl donor 16 µM *S*-adenosylmethionine *plus* 10 µCi/ml of [*methyl*-3H] *S*-adenosylmethionine (GE Healthcare; specific activity 70–80 Ci/mmol). The reaction mixture was incubated for 1 hour at 37°C and the reaction was stopped at 60°C for 30 min after addition of 1% SDS and 250 µg/ml of proteinase K. The incorporation of labeled methyl groups was evaluated on purified DNA in a Beckman LS-6800 liquid scintillation spectrometer.

### Genomic bisulphite sequencing

Briefly, genomic DNA (1 µg) was denatured by adding NaOH to a final concentration of 0.3 M for 15 minutes at 37°C. For the sulphonation reaction, the sample was incubated in the dark for 17 hours at 55°C in the presence of 3.1 M sodium bisulphite, 0.5 mM hydroquinone and 6.25 M urea in a final volume of 0.24 ml at pH 5.0. DNA was then purified from the reactions mixtures using the Wizard DNA Clean-Up system (Promega) and resuspended in 50 µl of water. Alkaline desulphonation of DNA was performed at 37°C for 15 min by the addition of NaOH to the final concentration of 0.3 M. This solution was neutralized by adding ammonium acetate (pH 7.0) to the a final concentration of 3.0 M. After ethanol precipitation, the modified DNA was dissolved in 20 µl of water.

Genomic sequencing analysis of *Dnmt*1 promoter region (Kimura et al. 2003) spanning from −364 to −103 (bp from the first codon) was performed on bisulphite modified genomic DNA (100 ng). The bisulphite modified promoter of *Dnmt*1 was amplified using the following primers: 5′- GGATTTTTTGGAAGTGGAATTATAG -3′ sense and 5′- CCACACAAAACAAAAAAATAAAAAAA -3′antisense. The amplified DNAs were purified and cloned into the TOPO TA-cloning vector (pCR 2.1-TOPO kit, Invitrogen). Twenty independent clones for each sample were cultured in LB medium and the corresponding recombinant plasmids were extracted (Fast Plasmid Extraction Kit, Eppendorf). The purified plasmids were directly sequenced on both strands by using a Rhodamina Terminator Cycle Sequencing Ready Reaction Kit (Applied Biosystem) and an ABI PRISM 310 DNA Sequencer (Applied Biosystem), Primer pairs for sequencing were M13 forward (−20) and M13 reverse included in the kit.

### Co-immunoprecipitation (Co-IP)

Nuclei obtained from L929 cells were lysed in IP buffer (50 mM Tris-HCl pH 7.5, 5 mM EDTA, 300 mM NaCl, 1% Nonidet P-40, 1% Triton X-100) supplemented with protease-inhibitors (complete EDTA-free, Roche Applied Science). Lysates (1.5 mg) were pre-cleared with protein A-(for IP anti PARP-1) or G (for IP anti Dnmt1)-agarose beads (Upstate) on a rotative shaker at 4°C for 2 h and 30 min. Pre-cleared lysates were incubated with specific antibodies (mouse monoclonal Ab anti-Dnmt1, Imgenex; and rabbit polyclonal Ab anti-PARP-1, Alexis) and with normal rabbit or mouse IgG (Santa Cruz Biotechnology) on a rotative shaker at 4°C. The agarose beads, previously saturated with bovine serum albumin (1 µg/µl) overnight, were added to the lysate/Ab solutions and incubated for 2 h on a rotative shaker at 4°C. Subsequently, beads were washed in IP buffer and boiled in SDS-PAGE sample buffer, and the eluted proteins were analyzed by western blotting.

### siRNA treatment

siRNA treatment was performed to knockdown Ctcf expression in L929 cells. SMART pool siRNAs (Dharmacon) specific for murine Ctcf were transfected into 0.16×10^6^ cells in 35 mm culture dishes using Lipofectamine 2000 (invitrogen). siGENOME non-targeting siRNAs (Dharmacon) were transfected as a negative control. After 48 hours from transfection cells were harvested and processed for western blot analysis.

### Methylation-sensitive southern blot analysis

DNA preparations (2 µg) were digested with 40 units of MspI or HpaII (New England Biolabs) restriction enzymes for 16 hours at 37°C. After 1.5% agarose gel electrophoresis, the digested DNA were blotted on Hybond-N nylon membrane (Amersham Biosciences), and the presence of new HpaII cutting sites was evidenced by hybridization to a 3′-digoxigenin-labeled single strand synthetic oligonucleotide as probe. Labeling of probe and detection was performed using digoxigenin oligonucleotide 3′-end labeling kit and digoxigenin luminescent detection kit (Roche Applied Science). Sequence of probe for minor satellite repeats was: 5′-GGAAACATGATAAAAACCACAGTGTAGAACATATTAGATGAGTGAGTTACACTGAA AAACACATTCGTTGGAAACGGGATTTGTAGAACAGTGTATATCAATGAGTTACAATGAGAAACATC- 3′ [49]. The oligo was made by custom primers synthesis service (Invitrogen). As positive control for DNA demethylation, digestion was performed in parallel on DNA from 5-AZA treated cells.

## Supporting Information

Materials and Methods S1 Supporting Material and Methods
(0.03 MB DOC)

Figure S1 Survival and cytotoxicity after PARG over-expression. A, Trypan blue exclusion test to determine the number of surviving cells after transfection of pCS2-Myc-PARG at 24 and 72 hours of puromycin selection, as compared to control cells. B, LDH assay to determine the relative cytotoxicity of transient transfection at 24, 48 and 72 hours post transfection (p.t.) of pCS2-Myc-PARG vs control cells. The value for the untreated samples was set at 1.0. Untreated: non-transfected cell; mock: cells transfected in absence of DNA. Data in A and B are reported as mean±S.E. of three independent experiments.
(7.74 MB TIF)

Figure S2 Analysis of cell cycle after PARG over-expression. Cell cycle progression at 24 and 72 hours of puromycin selection of cultures transfected with pCS2 or pCS2-Myc-PARG vectors assayed by cytofluorimetric analysis.
(1.57 MB TIF)
