# Supplementary material for: Parp1 Localizes within the Dnmt1 Promoter and Protects Its Unmethylated State by Its Enzymatic Activity
Source: PLoS One. 2009 Mar 5;4(3):e4717. doi: 10.1371/journal.pone.0004717 (PMC2650799; doi:10.1371/journal.pone.0004717)
Supplement: Materials and Methods S1 — Supporting Material and Methods (0.03 MB DOC) [file pone.0004717.s001.doc]

**Supporting Information**

**Supporting material and methods S1**

**Cell viability, cell-cycle analysis and** **cytotoxicity determination**

Cell number and viability were determined by direct Burker chamber counting of trypsinized and PBS-washed cells after trypan blue (Invitrogen) staining. Thereafter, cell cycle distribution was evaluated in each sample by acrydine orange (Sigma) staining followed by cytofluorimetric analysis. The percent of cells in G1, S, G2M cell cycle phases were determined by measuring simultaneously the DNA and RNA content. Modified FACScan equipment (Becton Dickinson) was used to measure fluorescence upon excitation at 488 nm. Five thousand cells were measured for each analysis at separate wavelength bands for DNA (green) and RNA (red). Samples were analyzed using Becton Dickinson software ModFit and CellQuest.

Cytotoxicity was evaluated by assaying Lactate Dehydrogenase (Korzeniewski and Callewaert 1983). Culture media (50 μl) were assayed on 96 well plates (Greiner bio-one). Absorption was measured after 15 minutes at 490 nm in an ELISA reader (AutoReader III, Ortho). A positive control of 100% cytotoxicity (1%Triton X-100 solution in the assay medium lyses the cells completely) was included in the assay. Background values from wells without cells were subtracted and average values for the triplicates calculated. Cytotoxicity within each sample was calculated as percent of sample absorbtion relative to the absorption of the each positive control.

Korzeniewski C, Callewaert DM (1983) An enzyme-release assay for natural cytotoxicity. Journal of immunological methods 64(3): 313-320.

**Supporting figure legends**

**Figure S1. Survival and cytotoxicity after PARG over-expression**

A, Trypan blue exclusion test to determine the number of surviving cells after transfection of pCS2-Myc-PARG at 24 and 72 hours of puromycin selection, as compared to control cells. B, LDH assay to determine the relative cytotoxicity of transient transfection at 24, 48 and 72 hours post transfection (p.t.) of pCS2-Myc-PARG *vs* control cells. The value for the untreated samples was set at 1.0. Untreated: non-transfected cell; mock: cells transfected in absence of DNA. Data in A and B are reported as mean ± S.E. of three independent experiments.

**Figure S2. Analysis of cell cycle after PARG over-expression**

Cell cycle progression at 24 and 72 hours of puromycin selection of cultures transfected with pCS2 or pCS2-Myc-PARG vectors assayed by cytofluorimetric analysis.
