# Supplementary figures and images for: Parp1 Localizes within the Dnmt1 Promoter and Protects Its Unmethylated State by Its Enzymatic Activity
Source: PLoS One. 2009 Mar 5;4(3):e4717. doi: 10.1371/journal.pone.0004717 (PMC2650799; doi:10.1371/journal.pone.0004717)

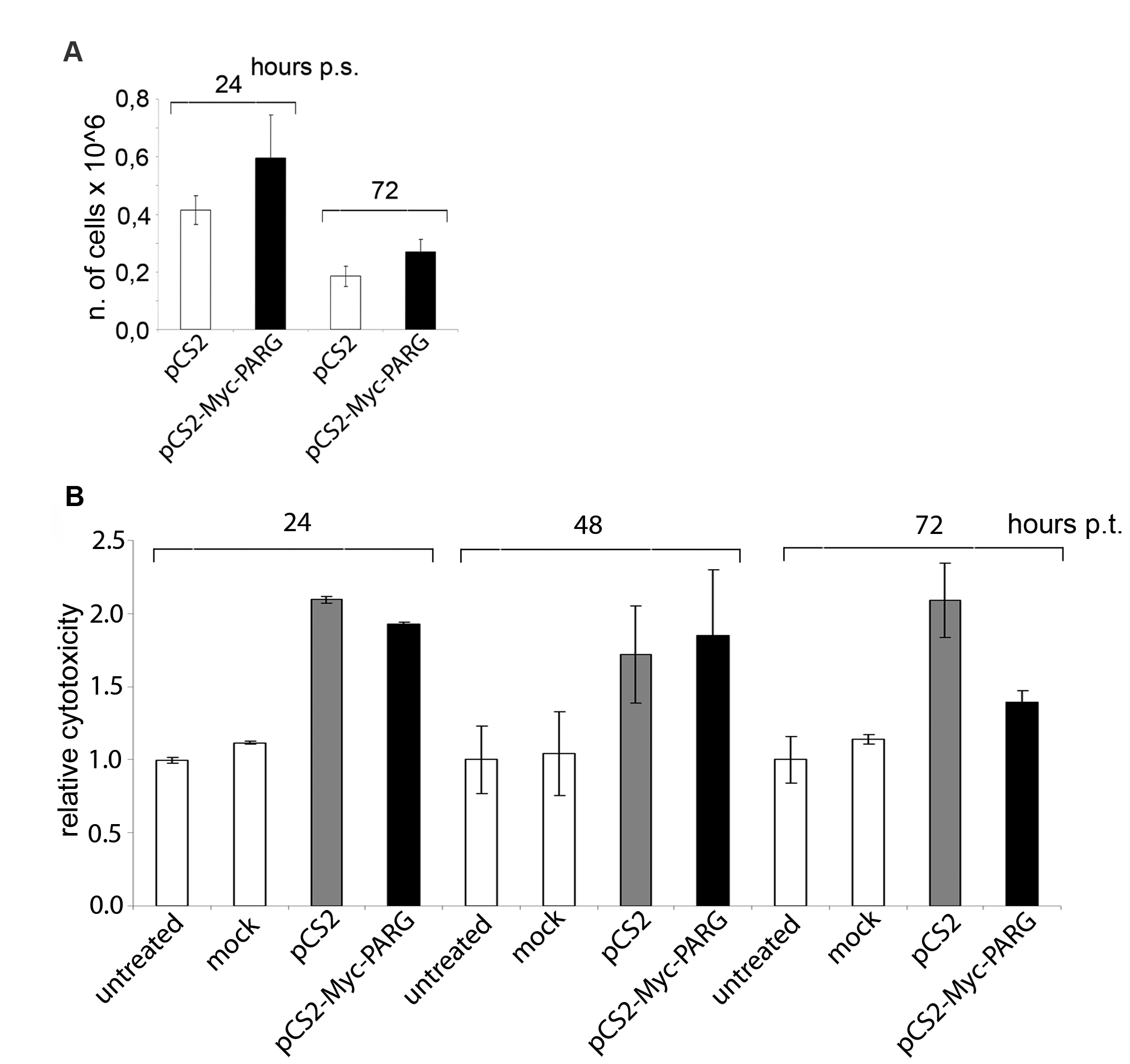

Supplement: Figure S1 — Survival and cytotoxicity after PARG over-expression. A, Trypan blue exclusion test to determine the number of surviving cells after transfection of pCS2-Myc-PARG at 24 and 72 hours of puromycin selection, as compared to control cells. B, LDH assay to determine the relative cytotoxicity of transient transfection at 24, 48 and 72 hours post transfection (p.t.) of pCS2-Myc-PARG vs control cells. The value for the untreated samples was set at 1.0. Untreated: non-transfected cell; mock: cells transfected in absence of DNA. Data in A and B are reported as mean±S.E. of three independent experiments. (7.74 MB TIF) [file pone.0004717.s002.tif]

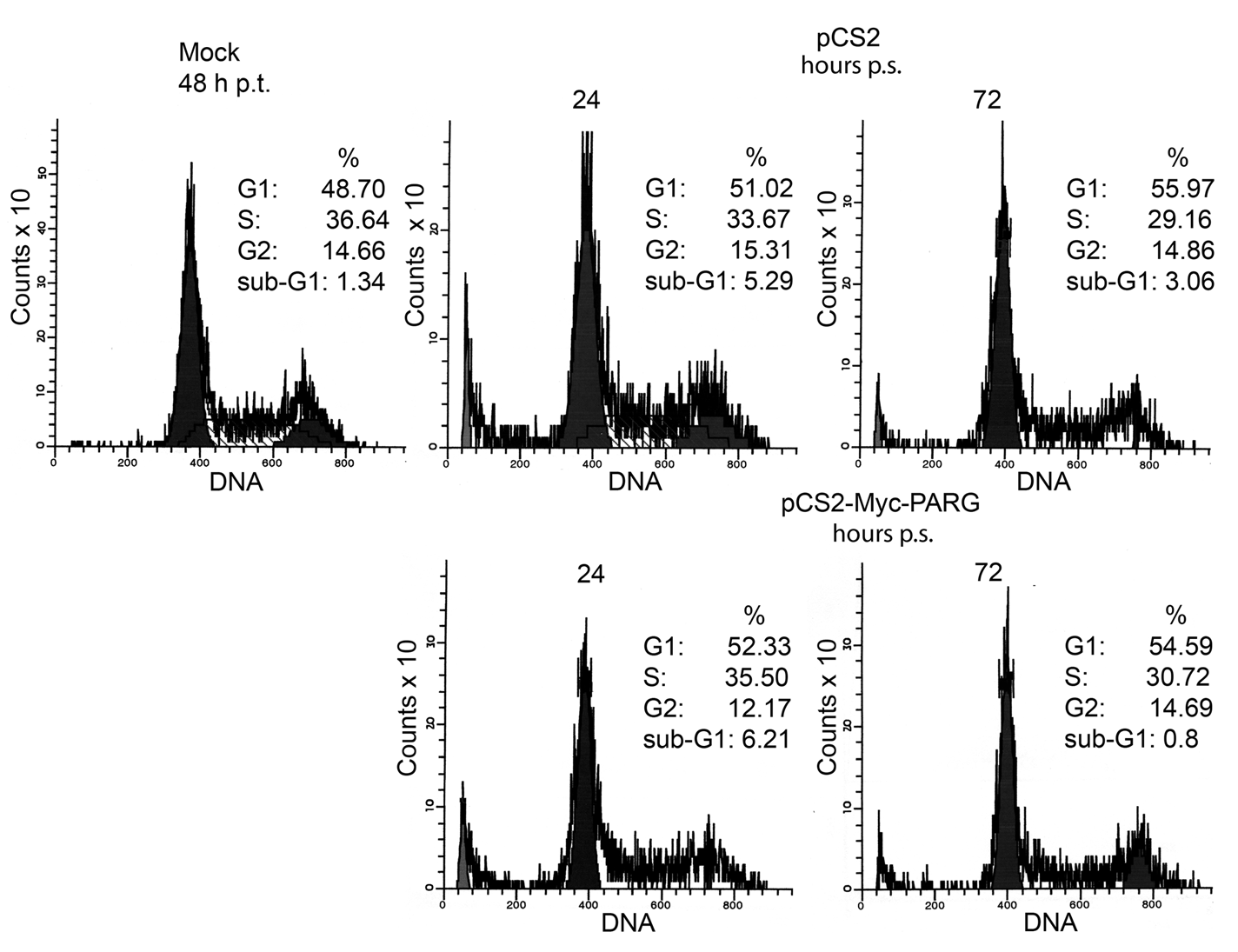

Supplement: Figure S2 — Analysis of cell cycle after PARG over-expression. Cell cycle progression at 24 and 72 hours of puromycin selection of cultures transfected with pCS2 or pCS2-Myc-PARG vectors assayed by cytofluorimetric analysis. (1.57 MB TIF) [file pone.0004717.s003.tif]
